# Single-unit data for sensory neuroscience: Responses from the auditory nerve of young-adult and aging gerbils

**DOI:** 10.1038/s41597-024-03259-3

**Published:** 2024-04-22

**Authors:** Amarins N. Heeringa

**Affiliations:** Research Centre Neurosensory Science and Cluster of Excellence “Hearing4all”, Department of Neuroscience, School of Medicine and Health Science, Carl von Ossietzky University Oldenburg, Carl von Ossietzky Straße 9-11, 26129 Oldenburg, Germany

**Keywords:** Cochlea, Sensory processing, Ageing

## Abstract

This dataset was collected to study the functional consequences of age-related hearing loss for the auditory nerve, which carries acoustic information from the periphery to the central auditory system. Using high-impedance glass electrodes, raw voltage traces and spike times were recorded from more than one thousand single fibres of the auditory nerve of young-adult, middle-aged, and old Mongolian gerbils raised in a quiet environment. The dataset contains not only responses to simple acoustic stimuli to characterize the fibres, but also to more complex stimuli, such as speech logatomes in background noise and Schroeder-phase stimuli. A software toolbox is provided to search through the dataset, to plot various analysed outcomes, and to give insight into the analyses. This dataset may serve as a valuable resource to test further hypotheses about age-related hearing loss. Additionally, it can aid in optimizing available computational models of the auditory system, which can contribute to, or eventually even fully replace, animal experiments.

## Background & Summary

Age-related hearing loss is one of the most prevalent diseases world-wide, as it affects over 65% of adults above 60 years of age^1,2^. Moreover, it is predicted to become increasingly more prevalent as our society ages. Elderly with age-related hearing loss often experience a reduced ability to communicate in daily settings. By affecting mental health, physical health, and social functioning, age-related hearing loss can result in social isolation and a decreased quality of life^3^. Moreover, age-related hearing loss is associated with an increased risk of cognitive impairment and dementia^4,5^.

Both peripheral cochlear damage and a decline of central processes in the brain are thought to contribute to age-related hearing deficits^6,7^. To unravel the different contributions of peripheral and central age-related degeneration, we studied the functioning of the auditory nerve, the sole connection between the peripheral and central auditory systems. The general aim of this work was to determine the consequences of age-related hearing loss on single auditory nerve fibre spiking activity.

An attractive and well-studied animal model to address this aim is the quiet-aged Mongolian gerbil. In this animal model, noise-induced cochlear damage is minimized, and thus the effects of aging on cochlear functioning can be studied in isolation. A large body of previous research has revealed age-related cochlear and central auditory pathologies in the quiet-aged gerbil^8–11^. Furthermore, the gerbil possesses good low-frequency hearing, which is important to refer, for example, speech-encoding deficits to the human condition^12,13^. And finally, since the gerbil can be trained to behaviourally make auditory discriminations, our results have also been directly linked to behavioural consequences of age-related hearing loss^14,15^.

**Fig. 1:**
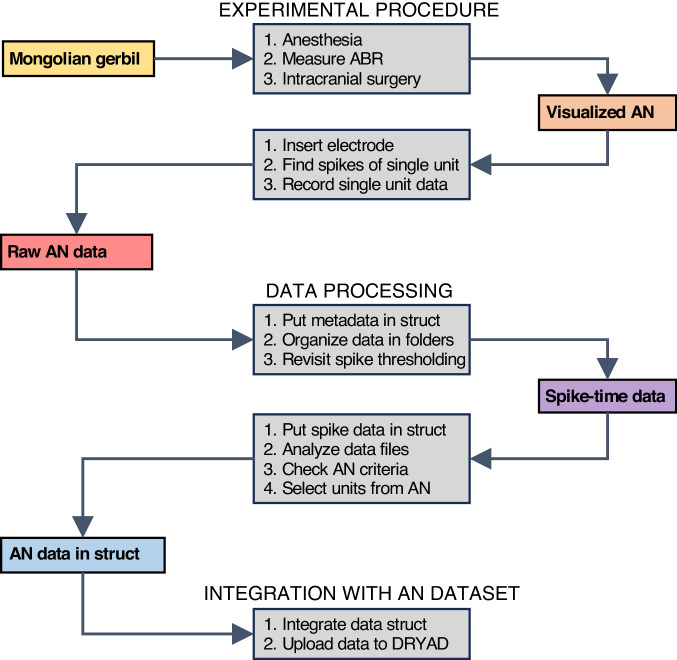
Workflow. This figure indicates how the data from one Mongolian gerbil were obtained and then processed. ABR, auditory brainstem response; AN, auditory nerve.

The dataset presented here contains the raw waveforms and spike times from single auditory nerve fibres of young-adult, middle-aged, and old gerbils, recorded while presenting a variety of acoustic stimuli to assess the functioning of the single fibres^16^. Gerbils were anaesthetized with ketamine/xylazine injections, and auditory brainstem responses (a type of compound response) were recorded to derive a measure of their general hearing sensitivity. The auditory nerve was approached dorsally through the cerebellum and a high-impedance glass electrode was slowly moved through the nerve. Single-unit recordings were made for as long as the surgical preparation was stable. Afterwards, spikes in the data were identified, data files were organized into folders for each single unit, data were analysed to characterize the fibre, and a set of criteria was used to ensure that the single unit had been isolated from the auditory nerve bundle. Figure 1 shows a schematic overview of the study.

The dataset contains a total of 1160 single-unit auditory nerve fibres, of which 314 were recorded in old gerbils (>36 months old) that had various degrees of age-related hearing loss. A gerbil of 36 months or older is vulnerable; approximately 50% of gerbils in our facility die before reaching this age. Furthermore, successful recordings of single auditory-nerve fibres were obtained in only 43% of the experimental old gerbils (compared to 88% in young adults). Common reasons were that they die early in the experiment due to unstable anaesthesia, leading to heart failure, or they had lost all their hearing sensitivity, and single fibres did not respond to acoustic stimulation. This increases the value of the recordings reported here, especially those from the oldest gerbils.

Outcomes from these data have been published before^14,15,17–20^. By openly sharing these data in combination with detailed metadata and start-up software, it can serve as a valuable resource to test hypotheses about age-related hearing loss that have not yet been addressed. Furthermore, the detailed description of the methods is aimed to improve reproducibility of the experiments, as well as to serve as a starting point for adding to this dataset. Data from animals, especially from those where the yield is small, should be used to the full, in an effort to reduce the number of animals sacrificed for scientific research. Finally, this dataset can aid in optimizing computational models, which may partly, or eventually even fully, replace animal experiments.

## Methods

### Animals

The dataset was collected from a total of 104 Mongolian gerbils, *Meriones unguiculatus*, of either sex that were born and raised in the animal facility at the Carl von Ossietzky Universität Oldenburg, Germany. The founder animals of this in-house colony came from Charles River Laboratories in 2009. Animals were group housed, kept on a 12:12 h light:dark cycle, fed *ad libitum*, and provided with cage enrichment. Average sound levels in the housing rooms were 48 and 55 dB A outside and during working hours, respectively. Sound levels were intentionally kept low, to minimize the effects of external, noise-related damage to the auditory system. Thus, the isolated effects of aging and age-related hearing loss on single auditory-nerve responses could be studied. Experimental procedures were in accordance with the ethics authorities of Lower Saxony, Germany, under the permit numbers AZ 33.9-42502-04-11/0337, AZ 33.19-42502-04-15/1990, and AZ 33.19-42502-04-21/3695.

### Surgical procedures

#### Anaesthesia

Initial anaesthesia of the gerbils was accomplished by intraperitoneal injection of ketamine (135 mg/kg; Ketamin 10%, bela-pharm GmbH or Ketamidor, WDT) and xylazine (6 mg/kg; Xylazin 2%, Ceva Tiergesundheit GmbH or Xylazin, Serumwerk) diluted in saline (0.9% NaCl). Anaesthesia was maintained by hourly subcutaneous injections with one third of the initial dose of the same mixture (45 mg/kg ketamine and 2 mg/kg xylazine). Additionally, if a hind-paw reflex was detected, an additional one sixth of the initial dose was injected. Meloxicam, a non-steroidal antiphlogistic agent (1 mg/kg; Metacam 2 mg/ml, Boehringer Ingelheim) was injected at the beginning of the experiment when the animal was sensitive to the surgical procedures. In some experiments, a lidocaine ointment (Xylocain Gel 2%, Aspen Pharma Trading Limited) was applied topically on the muscles overlying the place of craniotomy as an additional local analgesic. Anaesthetic depth was constantly monitored by electrocardiogram recordings using intramuscular needle electrodes in the front leg and the contralateral hind leg (DAM50, World Precision Instruments), and visualized on an oscilloscope (SDS 1102CNL, SIGLENT Technologies). Body temperature was monitored via a rectal probe and maintained at 38 °C by a homeothermic blanket (Harvard Apparatus). To avoid airway obstruction during the experiment, some middle-aged and old gerbils were tracheotomized, but breathed unaided. Of the total of 104 gerbils, 78 received additional oxygen (flow 1.5 l/min) in front of the tracheotomy or snout throughout the experiment. For each animal, details of the anaesthesia are specified in the metadata of the experiment (‘*exp.info.anesthesia*’).

#### Placement of the sound system

The head of the animal was fixed in a bite bar (Kopf Instruments, Tujunga CA, USA), with the head mount, in addition, fixed to the exposed frontal skull using dental cement. A small opening in the bulla was made to prevent build-up of negative pressure in the middle-ear cavity. The pinna was removed to expose the bony ear canal. Subsequently, the ear bar, which contained the speaker and calibration microphone, was placed directly onto the bony ear canal, and sealed using petroleum jelly. To avoid damage to the tympanic membrane, the diameter of the ear bar’s front end was slightly larger than the ear canal. For the current dataset, we used two different sound systems: either an ER-2 speaker (Etymotic Research, Inc.) in combination with a Knowles microphone (FG-23329, Knowles Electronics), or a Sennheiser speaker (IE-800, Sennheiser) in combination with an Etymotic microphone (ER7-c, Etymotic Research). The sound system that was used is specified in the metadata of each experiment (‘*exp.info.sound_system*’).

#### Accessing the auditory nerve

To access the auditory nerve, a craniotomy over the right cerebellum was carried out by carefully removing parts of the occipital, parietal, and temporal bones. Following a duratomy, cerebellar tissue was slowly aspirated until the brainstem was exposed. To expose the auditory nerve, a few small balls of paper tissue (<0.5 mm), drenched in saline, were placed between the temporal bone and the brainstem.

### Auditory brainstem response

#### Stimulus generation

Auditory brainstem responses (ABRs) were used to determine general hearing sensitivity and to monitor cochlear health during the single-unit recordings. ABRs were measured during the presentation of custom-generated chirps (0.3–19 kHz, 4.2-ms duration, 5-dB step size, 200–500 repetitions), to compensate for the frequency-dependent travelling wave delay in the gerbil cochlea^21,22^. Chirps were generated in MATLAB (version R2015b; The MathWorks, Inc., Natick, Massachussets, United States) and were calibrated and equalized using the most recently obtained calibration file. After each (re)placement of the ear bar, the calibration file was acquired by measuring sound pressure level (SPL) near the eardrum with the miniature microphone sealed in the same ear bar and the output amplified by a microphone amplifier (MA3, Tucker Davis Technologies [TDT]). Stimuli were presented through an external audio card (Hammerfall DSP Multiface II, RME Audio; 48 kHz sampling rate), amplified (HB7, TDT), and presented through the small speaker sealed into the ear bar.

#### Waveform recording

To record the ABR, platinum needle electrodes were placed subdermally ventral to the ear canal, and in the ipsilateral neck muscle for recording and referencing, respectively. The output of the needle electrodes was fed into an amplifier (1,000x amplification, 0.3–3 kHz bandpass filter; ISO-80, World Precision Instruments) and recorded using the external RME audio card. Custom-written MATLAB software (R2015b) averaged and stored the ABR waveforms across stimulus levels. ABR thresholds were defined as the lowest level that evoked clear ABR waves and a wave I amplitude >4 µV. The stimuli and thresholds of the ABR are specified in the metadata of each experiment (‘*exp.info.ABR*’).

### Recording of single unit auditory nerve fibres

After visualizing the auditory nerve bundle, single units were recorded using glass micropipette electrodes (GB120F-10, Science Products GmbH) pulled on a P-2000 electrode puller (Sutter Instruments Co.). Electrodes were filled with a high concentration potassium-chloride solution (3M-KCl) and had an impedance between 5 and 50 MΩ. After the electrode was mounted in the holder, it was manually positioned just above the auditory nerve bundle using a micromanipulator (Märzhäuser). The electrode holder was attached to an inchworm motor (IW-711, Burleigh, Inc.), which could be controlled remotely via a piezo microdriver and handset (6000 ULN and 6005 ULN handset, Burleigh). An Ag/AgCl pellet electrode (Warner Instruments) served as an electrical reference. Electrical signals were amplified (10x; WPI 767, World Precision Instruments), filtered (50/60 Hz; Hum Bug, Quest Scientific), made audible through a speaker (MS2, TDT), visualized on an oscilloscope (SDS 1102CNL, SIGLENT Technologies), digitized (RX6, TDT; 48,828 Hz sampling rate), and displayed in a graphical user interface (GUI) on a personal computer using custom-written MATLAB software (R2015b). While a broad-band noise search stimulus (50–70 dB SPL) was played through the in-ear speaker, the electrode was slowly advanced through the auditory nerve (1–5 µm step size), until spikes were seen on the oscilloscope and/or heard through the MS2 speaker. The hardware used to record single-unit auditory nerve fibres was kept the same throughout all experiments and is specified in the metadata of each experiment (‘*exp.data.recording_system*’).

### Data acquisition

#### Stimuli to characterize the auditory nerve fibre

After spikes were observed that preferably also responded to the broad-band noise search stimulus, a quick audio-visual estimate of the fibre’s best frequency (BF) and threshold were obtained, using online sliders of tone-burst frequency and level in the software’s GUI. The response range estimates were based purely on audio-visual cues and no spike-rate criterion was initially applied. Next, tone bursts with a frequency ranging from well below to well above the audio-visually estimated BF were presented (~1.5 octaves wide) at around 10 dB above the audio-visually estimated threshold, with a step size varying between 50–250 Hz, depending on the frequency range. These data are stored in the field ‘*exp.data.BF*’. The unit’s BF was defined during the experiment from the frequency-response curve as the tone frequency with the highest spike rate. Next, to obtain the unit’s rate-level function (RLF), tone bursts at BF were presented at a range of levels. These data are stored in the field ‘*exp.data.RLF*’. Depending on how stable the recording was, and on the research question for the experiment, data were also recorded while varying both the tone’s frequency and level to determine the fibre’s response field, tuning curve, and characteristic frequency (CF) (‘*exp.data.CF*’), while presenting tones of various levels at the best frequency with more repetitions (‘*exp.data.PH*’), while presenting clicks (‘*exp.data.CLICK*’), and in silence (‘*exp.data.SR*’). Methods and criteria to further calculate the unit’s response characteristics, such as BF, threshold, CF, spontaneous rate, phase locking, and latency, are described below (in the section ‘Data analysis for technical validation’).

Except for the clicks, all stimuli were calibrated using custom-made MATLAB software (R2015b) according to the latest calibration file. A new calibration file was obtained after each (re)placement of the ear bar, using the miniature microphone sealed in the ear bar near the speaker. 2-sample condensation clicks are ~97 dB pe (peak equivalent) SPL when presented with 20 dB attenuation, which was the default setting in these click recordings. The attenuation of the click can be found in the metadata of each click recording (‘*exp.data.CLICK.curvesettings*’). Metadata of tone bursts, such as acquisition duration, stimulus delay, stimulus ramps, and randomization, can be found in the metadata of the respective recording (e.g., ‘*exp.data.BF.curvesettings’*). The Data Structure document (‘*Data_Structure.pdf*’), published along with the dataset^16^, can be consulted for detailed descriptions on the variables stored in this field.

#### Complex acoustic stimuli

In addition to the recordings to characterize the auditory nerve fibre, many experiments also included auditory nerve fibre responses to complex acoustic stimuli. Briefly, in 21 of 104 experiments, responses to two 1-s noise bursts were recorded, where the second burst of each stimulus pair was 180° phase-inverted relative to the first burst (60 frozen repetitions). Responses to these noise bursts were used to study the effects of age-related hearing loss on single fibre temporal coding^17^. Next, in 5 of 104 experiments, responses to Schroeder-phase harmonic tone complexes with various duty cycles, sweep directions, and velocities were recorded^14^. In 17 of 104 experiments, responses to consonant-vowel-consonant logatomes were recorded. These responses were used to study vowel discrimination and representation in single fibres of young-adult and quiet-aged gerbils^15,19^. These complex acoustic stimuli were presented as .wav files; the waveforms, sampling rates, and number of samples are included in the dataset (‘exp.data.*NOISE/SPS/CVC*.acoustic_stimulus’). Furthermore, the metadata sheet (‘*metadata.csv*’), published along with the dataset^16^, indicates in which experiment these stimuli were presented. In addition, single-fibre responses to other complex acoustic stimuli were also obtained from these experiments, including responses to vowel-consonant-vowel logatomes in quiet and in noise, responses to TFS1 stimuli, which are sets of harmonic and inharmonic tone complexes that differ only in their temporal fine structure but not in their envelope, as developed by Moore & Sek^23^, and responses to amplitude-modulated tones in various levels of broadband noise with a spectral notch centred at the carrier frequency of the amplitude-modulated tone. The responses of these datasets are not yet fully analysed and will be uploaded and added to the full dataset as soon as the studies are published.

### Spike detection

During data collection, spike triggering was defined interactively. This allowed the researcher to estimate BFs and thresholds directly after collecting the data. However, spike amplitude often varied during the recording and the spike trigger could be difficult to track accurately online. Therefore, spike triggering was more carefully revisited offline by manually checking and adjusting the spike trigger trial-by-trial by a trained and experienced scientist. Raw waveforms were band-pass filtered (300–3000 Hz) with a 6^th^-order type II Chebyshev filter (*cheby2* MATLAB-function, 20-dB roll off). A manually set spike trigger threshold was applied to all trials based on visual inspection of the first five trials. Subsequently, each trial was carefully inspected, and the spike trigger was adjusted on a trial-by-trial basis whenever the trigger level was too low and included baseline activity or when the trigger level was too high and, as such, excluded spikes. Spike times were defined by the time of the peak in each waveform snippet that exceeded the set spike trigger. The metadata from this offline spike detection are stored in *‘exp.data.[recording type].curvesettings.analysis’*. The Data Structure document (‘*Data_Structure.pdf*’) can be consulted for detailed descriptions on the variables stored in this field. Note that the stored spike trigger applies to the filtered waveforms, as described above, and not to the raw waveforms as stored in the ‘*curveresp*’ fields. The resulting spike times are stored in *‘exp.data.[recording type].curvedata.spike_times’*.

### Data analysis for technical validation

The recordings for characterizing the auditory nerve fibre (recording types: BF [Best Frequency], CF [Characteristic Frequency], PH [PHase locking], CLICK, RLF [Rate-Level Function], and SR [Spontaneous Rate]) have been analysed to give the user a general idea of the auditory nerve fibre type and to search through the data more effectively. The outcomes of these analyses are stored along with the raw data in the data structs (‘*exp.data.[recording type].analysis*’) and are presented below under the section Technical Validation.

#### Calculating spike rate

For the analysis of recordings with responses to tones (BF, CF, PH, and RLF recordings), the tone-burst-evoked spike rate was calculated separately for each trial. The number of spikes that were recorded during the presentation of the tone, i.e. between t_1_ ( = stimulus onset) and t_2_ ( = stimulus onset + stimulus duration), were divided by the stimulus duration in s. Subsequently, spike rates were averaged over the number of repetitions of unique frequency-level combinations. The experimenter had the option of including silent trials interleaved between the tone-burst trials, presented as often as each unique frequency-level combination (defined as the number of repetitions). These silent trials can be used to estimate the unit’s spontaneous rate. When this option was included, spontaneous rates associated with these recordings (stored in ‘*exp.data.[recording type].analysis.sr*’) were always calculated over the total trial duration (as opposed to the stimulus duration) and averaged over the repetitions containing silent trials. By contrast, spontaneous rates stored in ‘*exp.data.SR.analysis.sr*’ were derived from longer trials (~ 2.4 s) and were also averaged over the repetitions. As the total recording time in silence of the SR recording type was longer than that of the silent trials in tonal recording types (~240 s in SR recordings vs. ~0.8 s in RLF recordings), the spontaneous rate estimate is likely to be the more precise for the SR recording type. For units without data in the ‘*SR*’ field, the author advises choosing the tonal recording type with the most repetitions, indicating the longest total time and, hence, the most reliable estimate of spontaneous rate. This is typically the PH recording (‘*exp.data.PH.analysis.sr*’), followed by the RLF recording (‘*exp.data.RLF.analysis.sr*’).

#### Analysis of responses to tone bursts

For BF recordings, the mean tone-evoked spike rate was plotted as a function of stimulus frequency. A smoothing spline function was fitted to the data and the peak of this fitted function stored as the best frequency in Hz (‘*exp.data.BF.analysis.bf*’), according to Aralla, *et al*.^24^. The frequency-response curve can be reconstructed by plotting the mean and standard deviation of the spike rates as a function of stimulus frequency, which are stored in ‘*exp.data.BF.analysis.rates*’, ‘*exp.data.BF.analysis.stdevs*’, and ‘*exp.data.BF.analysis.freqs*’, respectively. This analysis procedure was carried out by the function *BFextract_func.m*.

For RLF recordings, the mean tone-evoked spike rate was plotted as a function of stimulus level. The threshold was defined as the lowest stimulus level evoking a spike rate higher than 15 spikes/s and higher than [mean spontaneous rate + 1.2 times standard deviation spontaneous rate]. Spontaneous rate was determined from the silent trials of the same recording. The threshold was stored in ‘*exp.data.RLF.analysis.threshold*’. The rate-level function can be reconstructed by plotting the mean and standard deviation of the rates as a function of stimulus level, which are also stored in ‘*exp.data.RLF.analysis*’. The frequency of the tone burst corresponds to the best frequency as determined online during the experiment and is close, but often not exactly the same as the one stored in *exp.data.BF.analysis.bf*. The analysis procedures were carried out by the function *RLFextract_func.m*.

PH recordings are like RLF recordings, except that they contain fewer levels and more repetitions per level. As such, phase locking can be reliably studied per stimulus level. PH recordings were typically only collected for auditory nerve fibres with a relatively low best frequency (<~5 kHz). Vector strength (vs) was calculated as follows:

$$vs=\frac{1}{N}\left|\mathop{\sum }\limits_{j=1}^{N}{e}^{i\varphi \left(j\right)}\right|$$

where *N* is the total number of spikes and *φ(j)* is the phase of the *j*^th^ spike within the period of the tone. The vector strength is calculated based on spikes from all repetitions for each level and is stored as an array (‘*exp.data.PH.analysis.vs*’) along with the stimulus levels (‘*exp.data.PH.analysis.levels*’) and frequency (‘*exp.data.PH.analysis.frequency*’). The significance of the vector strength (vs) is determined by calculating a p-value as follows:

$$p={e}^{-N.v{s}^{2}}$$

When N < 50, the p-value is NaN (which stands for Not-a-Number, a value of a numeric data type that does not contain a number), also making the vector strength invalid. When p < 0.001, the vector strength is considered significant and meaningful. P-values corresponding to the vector strengths at each stimulus level are stored in ‘*exp.data.PH.analysis.prob*’. The analysis procedures were carried out by the function *PHextract_func.m*.

In CF recordings, responses were recorded to tone bursts while varying both tone frequency and level. For each stimulus frequency, the fibre’s rate threshold was defined as the lowest level with a spike rate higher than T = [mean spontaneous rate + 1.2 times standard deviation spontaneous rates]. Spontaneous rate was determined from the silent trials of the same recording. T could be manually adjusted when needed, for example for fibres with a spontaneous rate of 0 spikes/s. The tuning curve was constructed by plotting the rate threshold as a function of the stimulus frequency. Fibre threshold is defined as the lowest threshold of the tuning curve (‘*exp.data.CF.analysis.threshold*’) and the characteristic frequency (CF) as the stimulus frequency that gave rise to this threshold (‘*exp.data.CF.analysis.cf*’). The Q_10dB_ (‘*exp.data.CF.analysis.q10*’) was calculated by dividing the characteristic frequency by the tuning-curve bandwidth at 10 dB above threshold. Q_10dB_ is NaN (Not-a-Number) when this bandwidth could not reliably be established, for example when the frequency range was too narrow. The response field can be reconstructed by creating a 3D-surface plot of the mean rates as a function of stimulus frequency and stimulus level, which are also stored in ‘*exp.data.CF.analysis*’. The analysis procedures were carried out by the function *CFextract_func.m*.

#### Analysis of responses to clicks

From CLICK recordings, the response latency was determined using three different methods. A Poisson probability density function was constructed from the spikes that were evoked after the onset of the click, across all repetitions. The response latency was defined as the time when this function fell below a threshold of 10^−6^ ^25^ and was stored in ‘*exp.data.CLICK.analysis.latency_poisson*’. Response latency was also measured by the first incidence when two consecutive bins (0.05-ms bin size) in the peristimulus time histogram were higher than the highest bin before the onset of the click^26^. This value was stored in ‘*exp.data.CLICK.analysis.latency_2bins*’. Finally, the mean and median first-spike latency (FSL) were calculated and stored in ‘*exp.data.CLICK.analysis.fsl_mean*’ and ‘*exp.data.CLICK.analysis.fsl_median*’. Furthermore, the standard deviation (‘*exp.data.CLICK.analysis.fsl_std*’), variance (‘*exp.data.CLICK.analysis.fsl_var*’), and interquartile range (‘*exp.data.CLICK.analysis.fsl_iqr*’) of the first-spike latencies across the repetitions were stored as a measure of first-spike jitter. All click latencies are relative to the onset of the click. Analyses were carried out by the function *Clickextract_func.m*.

### Data analysis of complex stimuli

From NOISE, CVC, and SPS recordings, the number of trials that can be included in the analysis as well as the trial indices are stored in ‘*exp.data.[recording type].analysis.ntrials*’ and ‘*exp.data.[recording type].analysis.trials*’, respectively. Furthermore, the average rate response, calculated over 5-ms bins, along with the time vector of the bin centres, are stored in ‘*exp.data.[recording type].analysis.PSTH_rates*’ and ‘*exp.data.[recording type].analysis.PSTH_centers*’, respectively. The PSTH_rates and PSTH_centers variables can be used to plot the mean peri-stimulus time histogram (PSTH) of the recording.

## Data Records

### Repositories

All data files from individual animals (‘*G#.mat*’), as well as the data file containing all experiments (‘*all_AN_data.mat*’), the metadata sheet (‘*metadata.csv*’), and the document describing all fields in the structure (‘*Data_Structure.pdf*’), are shared on the DRYAD server^16^. The data can easily be downloaded without restrictions and are shared under Creative Commons 0 (CC0). The software toolbox is shared on the Zenodo server (10.5281/zenodo.10370064), is linked to the dataset on DRYAD, and is licensed under the GNU General Public Licence (GPL)^16^. The full dataset is also available as the function data_heeringa2024 in the Auditory Modeling Toolbox (AMT) version 1.6^27^.

### Raw data from individual animals

**Fig. 2:**
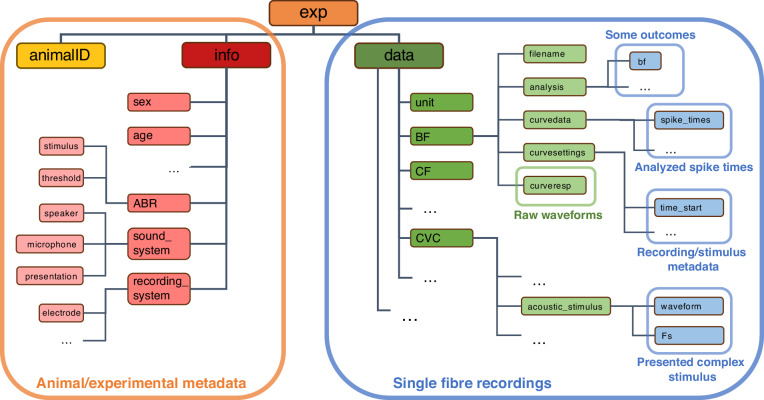
Hierarchical organization of a data structure file. Overview of the location of each data- and metadata type within the data structure.

Data were stored in a nested MATLAB structure (type struct) to preserve the organization between the raw data and the associated metadata. For each animal, one struct was made with a standard variable name ‘*exp*’, which was saved as a .mat-file. Figure 2 shows an overview of the hierarchy in the struct and the location of the different kinds of metadata, outcomes, spike times, and raw waveforms. In the first layer of the struct, there are three fields: 1) ‘*exp.animalID*’, a string with the unique ID of the experiment, which is similar to the name of the data file, 2) ‘*exp.info*’, a struct with all metadata relevant to the experiment, e.g., the animal’s sex, age, and hearing threshold, but also the sound and recording systems that were used, 3) ‘*exp.data*’, a struct with the data that were recorded from the single auditory nerve fibres.

The single-unit data are organized as follows. All data recorded from one fibre were stored in one row (*#*) in the struct (‘*exp.data(#)*’). Each fibre has a unique name within that animal, stored in ‘*exp.data(#).unit*’. All fibres have the same fields, that can be either filled or empty depending on whether the recording was obtained from that given fibre. Within a filled data field, e.g. ‘*exp.data(#).BF*’, there are again five fields: 1) ‘*exp.data(#).BF.filename*’, a string with the original filename, 2) ‘*exp.data(#).BF.analysis*’, a struct containing analysed outcomes of the recording, 3) ‘*exp.data(#).BF.curvedata*’, a struct that stores the spike times and the variables for each trial, 4) ‘*exp.data(#).BF.curvesettings*’, a struct that stores all metadata relevant to the recording, 5) ‘*exp.data(#).BF.curveresp*’, a struct that stores the raw recorded voltage traces for each trial. Each entry in this structure is described in detail in the document ‘*Data_Structure.pdf*’.

For each individual gerbil (also called an experiment in this context), there is a single .mat file containing all associated raw data. To keep file size within workable limits of < 1 Gb, data from a few experiments (n = 3 at time of publication) were separated into two or three different .mat files. These can be recognized by the ‘*_#*’ after the animal ID in the filename, where *#* is the sequence number. While the metadata of the experiment are the same between the different files from one experiment (‘*exp.animalID*’ and ‘*exp.info*’), the data of the single fibre recordings are different between the sequence numbers (‘*exp.data*’).

### Files that help users search through the dataset

There is one .mat file that contains all experiments in one struct (‘*all_AN_data.mat*’). When this file is loaded into MATLAB, the variable is named ‘*all_exp*’ and the experiments are all listed consecutively with their corresponding animalID, info, and data fields. In this struct, the raw waveforms (‘*curveresp*’) and, if available, the acoustic stimulus (‘*acoustic_stimulus*’) were deleted to keep the file size manageable. The struct can be easily searched through to find, for example, animals of a certain age, experiments in which certain stimuli were presented, or auditory nerve fibres with a certain range of best frequencies. Furthermore, this struct can be used to make a metadata sheet of the latest version of the dataset, using the code *check_dataset.m*.

**Fig. 3:**
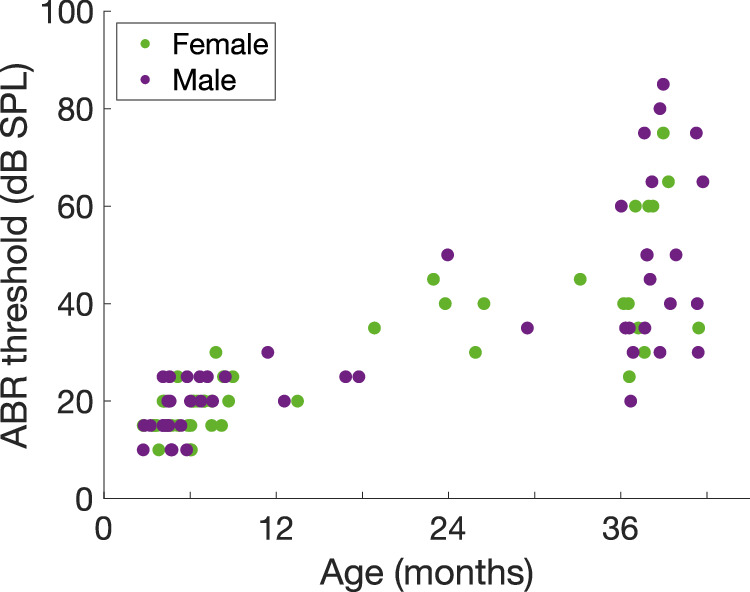
Hearing sensitivity of the gerbils. The auditory brainstem response (ABR) thresholds of females (green markers) and males (purple markers) in response to broadband chirps (0.3–19 kHz) as a function of their age in months.

The dataset consists of 104 experiments, with a total of 1160 single auditory nerve fibres. Table 1 lists the characteristics of the gerbils and the fibres in this dataset. Note the slightly skewed distribution of quiet-aged gerbils towards more males. This is mostly due to a high risk of ovarian cancer in older female gerbils, resulting in more early deaths before or during the experiments of females compared to males. To illustrate age-related hearing loss in both the male and female animals of this dataset, a rough estimate of hearing sensitivity, as determined by the auditory brainstem response (ABR) to chirps (0.3–19 kHz), is plotted as a function of the animal’s age (Fig. 3). Note the large variability in age-related hearing loss among the old animals, which is typical for the Mongolian gerbil^8^.

**Table 1:** Characteristics of the animals and auditory nerve fibres in the dataset.

	Young-adult gerbils (<12 months)	Middle-aged gerbils (12–36 months)	Quiet-aged gerbils (>36 months)
Number of animals (number of females)	60 (32)	13 (7)	31 (11)
Age in months, mean +/- SD (range)	5.5 +/− 2.0 (2.7–11.4)	22.5 +/− 6.2 (12.6–33.2)	38.3 +/− 1.7 (36.0–41.7)
ABR threshold in dB SPL, mean +/- SD (range)	18.1 +/− 5.3 (10.0–30.0)	34.2 +/− 10.2 (20.0–50.0)	49.0 +/− 18.0 (20.0–85.0)
Weight in grams, mean +/- SD (range)	74.6 +/− 11.1 (51.2–100.0)	83.4 +/− 12.7 (66.8–103.0)	86.4 +/− 11.6 (60.0–120.0)
Total number of single units	759	87	314
Range of best frequency in Hz	428–15,825	350–15,091	620–15,762

ABR: auditory brainstem response; SD: standard deviation; SPL: sound pressure level.

## Technical Validation

### Verification of single-unit recording and specificity to the auditory nerve bundle

**Fig. 4:**
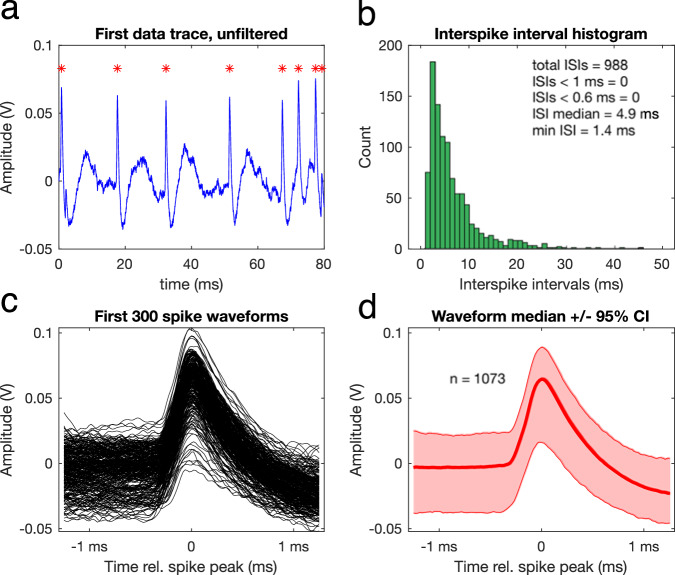
Inter-spike intervals and spike waveforms of an example recording. (**a**) The unfiltered, first data trace of the recording. Spike times are indicated with red asterisks. (**b**) The inter-spike interval (ISI) histogram, including descriptive statistics and the total number of short ISIs (<1 ms and <0.6 ms). (**c**) The waveforms of the first 300 spikes that were recorded. Time = 0 ms indicates the peak of the spike, i.e. spike time. Waveforms are plotted between −1.3 ms and +1.3 ms from the spike peak. (**d**) The median spike waveform (red line) with 95% confidence intervals (CI; shaded red). The number in the plot indicates the total number of spikes in the recording and used for this plot. This figure is the output of the function *checkAN.m*, of the BF recording of animalID ‘G220922’ and unit ‘3p_607’.

**Fig. 5:**
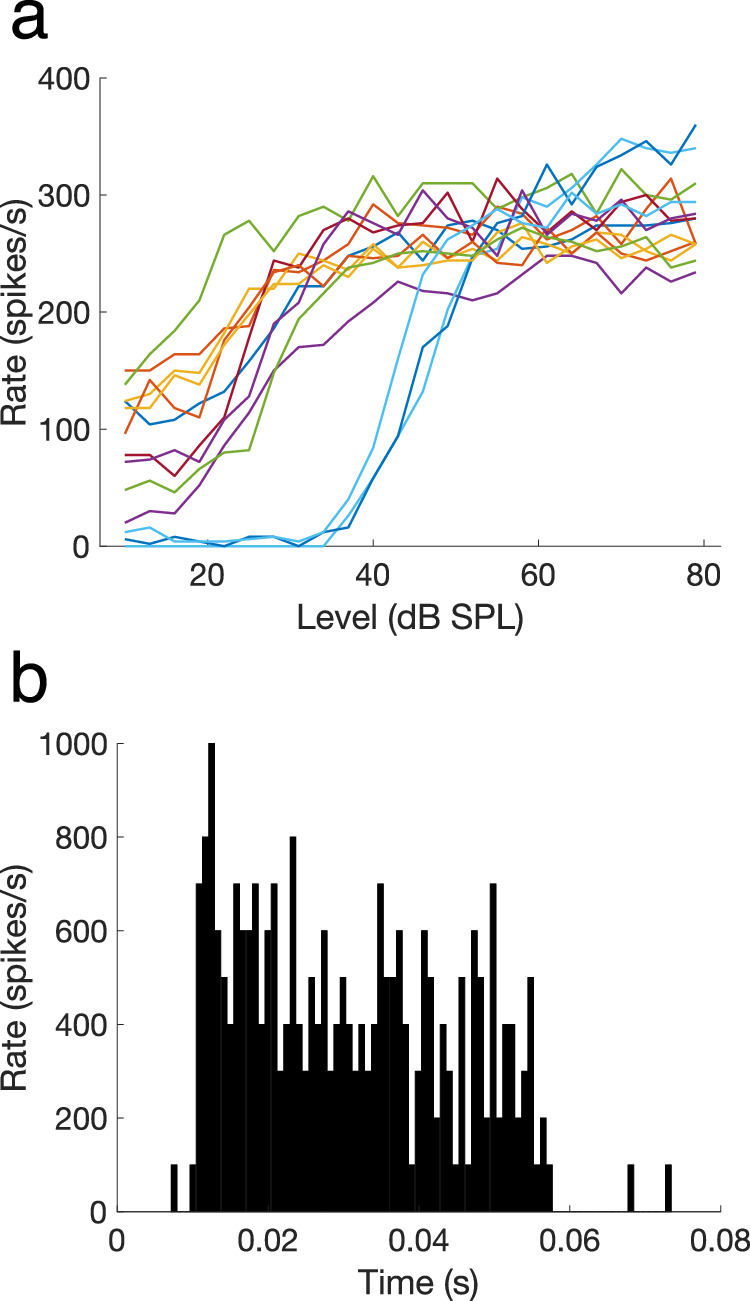
Criteria for specificity to the auditory nerve bundle. (**a**) Rate-level functions recorded from one gerbil (‘G220922’). Average firing rate is plotted as a function of stimulus level. Different colours indicate rate-level functions from different units. (**b**) An example of a peri-stimulus time histogram (PSTH) derived from tone burst responses at best frequency (animalID = ‘G220908’, unit = ‘3p_181’). Responses in the BF recording (level = 30 dB SPL, 5 repetitions) and the RLF recording (level = 40 dB SPL, 10 repetitions) are combined. This fibre had a best frequency of 7.6 kHz and a threshold at 19 dB SPL.

First, the recording was inspected for the possibility of a multi-unit recording, that is when spikes were derived from more than one fibre. Inter-spike intervals were assessed through the output of the function *checkAN.m* (see Fig. 4b). Units with multiple inter-spike intervals <0.6 ms, which is the absolute refractoriness of auditory nerve fibres^28^, were excluded from the dataset. We encountered this situation only rarely. Next, to ensure that the spikes derived from an auditory nerve fibre, and not from a neuron of the cochlear nucleus which can occasionally be encountered in the same general area of electrode placement, three criteria were used:

1. The median spike waveform across all spikes of one recording was carefully inspected for the presence of a prepotential, which would indicate that the spikes derived from a ventral cochlear nucleus bushy cell^29,30^. The function *checkAN.m* was used for this purpose, which plots one unfiltered trial of the recording (Fig. 4a), the inter-spike interval histogram (Fig. 4b), the first 300 spike waveforms (Fig. 4c), and the median spike waveform with 95% confidence intervals (Fig. 4d). We did not encounter any prepotentials in our recordings.
2. The shape of the rate-level function was carefully checked for atypical shapes. Typically, rate-level functions from the auditory nerve fall into one of the following three categories: straight, sloping saturating, or flat saturating^20,31,32^. When a rate-level function showed nonmonotonicity at levels lower than 80 dB SPL, it indicated that the spikes derived from a non-primary cell receiving inhibitory input. The unit was then excluded from the dataset. Figure 5a shows all rate-level functions recorded from one gerbil, with both flat-saturating and sloping-saturating shapes.
3. The responses to tones at 20 and 30 dB above threshold were examined for non-primary-like shapes. When the response was clearly non-primary like, the unit was excluded from the dataset. When available, data derived from BF-, RLF-, and PH-recordings were combined for this purpose. Figure 5b, constructed using the function *makePSTH.m*, shows a response shape that is typically encountered for auditory nerve fibres.

### Consistency with data from other labs

To further verify the reliability of the dataset, several outcomes of the analyses were plotted, such as best frequencies, thresholds, spontaneous rates, and phase locking metrics. Three age groups were defined: 1) young-adult gerbils, <12 months of age, 2) middle-aged gerbils, 12–36 months, and 3) old gerbils >36 months. These scatter plots and distributions were compared to auditory nerve data published for young-adult gerbils from other labs.

**Fig. 6:**
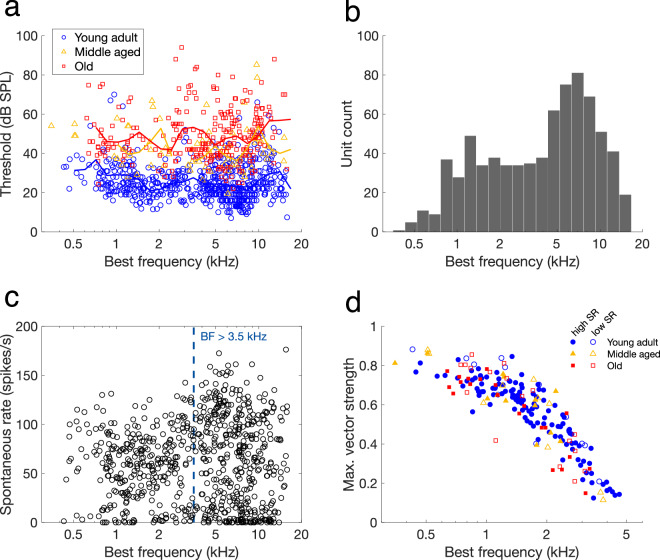
Physiological properties of the dataset. (**a**) Threshold plotted as a function of the fibre’s best frequency. Data from young-adult, middle-aged, and old gerbils are plotted in blue, yellow, and red markers, respectively. Solid lines represent the moving average for each age group. (**b**) Distribution of best frequencies of all fibres recorded in young-adult gerbils. (**c**) Spontaneous rate as a function of best frequency. The blue dashed line indicates the border between fibres with a low- and fibres with a high best frequency at 3.5 kHz. (**d**) Maximum vector strength in response to a tone at the fibre’s best frequency plotted as a function of best frequency for young-adult (blue circles), middle-aged (yellow triangles), and old gerbils (red squares). High-spontaneous rate (high-SR) and low-spontaneous rate (low-SR) fibres are plotted separately with filled and open markers, respectively, with 18 spikes/s as a cut-off rate^33^. Only vector strength values that were significant (p < 0.001) are plotted (see Methods).

Figure 6a shows the distribution of best frequencies and thresholds across the age groups. Fibres of young-adult gerbils exhibited two regions of best sensitivity (lowest thresholds), separated by a frequency region (around 3–4 kHz) with slightly less sensitive and fewer fibres. This is typical for the auditory nerve of the gerbil: it was observed and discussed in previous studies from four different labs^33–36^. Distributions of best frequencies and thresholds derived from middle-aged and old gerbils have not previously been published by other labs. The area with higher thresholds and fewer fibres separates the gerbil cochlea into a low- and a high-frequency region. For the young-adult gerbils in the current dataset, highest thresholds were at 3.5 kHz and fewest fibres were in the bin bordered by 2.5 and 3.0 kHz (Fig. 6b). This is consistent with the dataset of Huet, *et al*.^36^ and close to the border frequency of 4 kHz suggested by Ohlemiller and Echteler^34^ and by Müller^35^.

Figure 6c shows spontaneous rate plotted as a function of best frequency. Among the high-frequency fibres (>3.5 kHz), there was a cluster of fibres with low spontaneous rates. This is typical for the gerbil; it has been observed in previous studies from different labs^33,34,36^. The low-frequency fibres show a bimodal distribution of spontaneous rate, with a mode around 5 spikes/s and one around 60 spikes/s. This is also consistent with previously published distributions^33,34,36^.

Figure 6d shows the maximum vector strength in response to a best-frequency tone plotted as a function of the fibre’s best frequency. The maximum best frequency at which significant phase locking was recorded in young-adult gerbils was 4.6 kHz. This is consistent with previously published data from Versteegh, *et al*.^37^, who reported an upper phase-locking frequency for gerbil auditory nerve fibres of 4 to 5 kHz. Furthermore, the highest vector strength values were found among the fibres with low best frequencies (<1.5 kHz) and low spontaneous rates (<18 spikes/s, shown in open markers Fig. 6d). This is also consistent with previous work in gerbils and with vector strength recorded in auditory nerve fibres of cats^37,38^. Phase locking did not change in the aged animals^17^.

### Sampling across best frequency for the different recording types

**Fig. 7:**
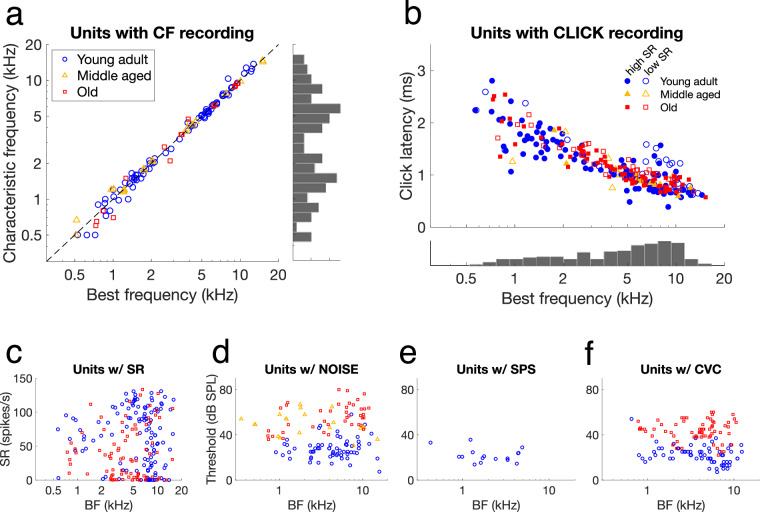
Sampling across the frequency axis. (**a**) For fibres for which a CF recording was obtained (n = 119), the characteristic frequency derived from the response field is plotted as a function of the fibre’s best frequency. A histogram of the characteristic frequencies is shown on the right. The black dashed line indicates y = x. (**b**) For fibres for which a CLICK recording was obtained (n = 261), the click latency, as determined by the 2-bin method, is plotted as a function of the fibre’s best frequency. High- and low-SR fibres are plotted separately in solid and open markers. A histogram of the best frequencies is shown below. (**c**) For fibres for which a SR recording was obtained (n = 203), the spontaneous rate (SR) derived from this recording is plotted as a function of the fibre’s best frequency (BF). The legend of panel (**a**) also applies here. (**d**–**f)** The sampling across BF and threshold for the complex stimuli recordings NOISE (n = 143, panel **d**), SPS (n = 22, panel **e**), and CVC (n = 135, panel **f**) in young-adult, middle-aged, and old gerbils. The legend of panel **(a)** also applies here.

Distribution of the recorded auditory nerve fibres across the frequency axis was plotted to illustrate the sampling of units within age groups for the different recording types (Fig. 7). BF distributions of two large datasets, CF and CLICK (Fig. 7a,b, respectively), are representative of the distribution of the full dataset (Fig. 6b). Furthermore, these figures confirm that the characteristic frequency derived from the CF recordings correlated strongly with the best frequency derived from the BF recordings, with no systematic deviation towards higher or lower frequencies (Fig. 7a). Click latency had a strong negative correlation to best frequency, illustrating the travelling wave delay along the cochlea (Fig. 7b). Sampling distribution across best frequency of the remaining datasets are shown in the lower panels (Fig. 7c–f). No SR, SPS, and CVC recordings were obtained in middle-aged gerbils, while SPS responses were only recorded in young-adult gerbils. Sampling across the best frequency range of RLF and PH recordings are shown in Fig. 6a and Fig. 6d, respectively.

## Usage Notes

### Code to help search through the dataset

Three main scripts are provided to help the user search through the full dataset, as well as within a struct of one animal.

1. *check_dataset_metadata.m* loops through the ‘*all_exp*’ struct and focusses on the metadata of the experiments. It recreates the metadata sheet (‘*metadata.csv*’), which can be used to select an animal of interest and investigate it further in *check_dataset_animal.m*. This script was used to generate Table 1 and Fig. 3.
2. *check_dataset_units.m* loops through the ‘*all_exp*’ struct and focusses on the analysed outcomes of the single units. It can be used to plot any of these outcomes against each other, typically with the fibre’s best frequency on the horizontal axis. This script was used to generate all panels of Figs. 6 and 7.
3. *check_dataset_animal.m* loops through the units of an ‘*exp*’ struct of one animal. It generates a scatterplot of the threshold as a function of the best frequency of all the fibres recorded in that animal and a plot with all rate-level functions of that animal in one graph. This script was used to generate Fig. 5a (from ‘G220922.mat’). It also calls the function *check_AN.m*, which plots the unfiltered first trial, the inter-spike interval histogram, the first 300 spike waveforms, and the median spike waveform +/− 95% confidence interval of a given recording. The output of this function is shown in Fig. 4 (BF recording of ‘G220922.mat’, unit ‘3p_607’ [i = 12]). The main script also calls the function *makePSTH.m*, which is used to generate a peri-stimulus time histogram (PSTH) of all responses to tone bursts at or close to a given stimulus level above the fibre’s threshold. *makePSTH.m* was used to generate Fig. 5b, based on the spike times of animalID ‘G220908’ from unit ‘3p_181’ (i = 23) at 20 dB above threshold (TestLevel = 20).

### Code to re-analyse the spike times for auditory nerve fibre characterization

The script *call_extract_func.m* calls the functions that were used to generate the analysed outcomes of one unit of one animal. These include the following functions:

1. *BFextract_func.m*, which generates the frequency-response curve to derive best frequency.
2. *CFextract_func.m*, which generates the receptive field and tuning curve to derive characteristic frequency, threshold, and Q_10dB_, a measure of frequency selectivity.
3. *PHextract_func.m*, which calculates vector strength and plots it as a function of stimulus level.
4. *CLICKextract_func.m*, which calculates click latency in three different ways and visually demonstrates the outcomes of each method.
5. *RLFextract_func.m*, which generates the rate-level function to derive threshold and rates at each stimulus level.
6. *SRextract_func.m*, which calculates the spontaneous rate from a long recording in silence.

## Data Availability

The code that is described at *Usage Notes*, along with all functions that are called by this code, can be accessed through Zenodo without restrictions (10.5281/zenodo.10370064)^16^. The GPL license enables re-users to run, study, share, and modify the software in any medium or format. Other researchers who use this dataset and code are encouraged to also share their code. The code will also be available within the Auditory Modeling Toolbox (AMT) version 1.6 as the function exp_heeringa2024^27^. All code was written in MATLAB and has been tested in version R2023b.
